# Author Correction: *JAK2V617F* mutation drives vascular resident macrophages toward a pathogenic phenotype and promotes dissecting aortic aneurysm

**DOI:** 10.1038/s41467-022-35645-z

**Published:** 2022-12-23

**Authors:** Rida Al-Rifai, Marie Vandestienne, Jean-Rémi Lavillegrand, Tristan Mirault, Julie Cornebise, Johanne Poisson, Ludivine Laurans, Bruno Esposito, Chloé James, Olivier Mansier, Pierre Hirsch, Fabrizia Favale, Rayan Braik, Camille Knosp, Jose Vilar, Giuseppe Rizzo, Alma Zernecke, Antoine-Emmanuel Saliba, Alain Tedgui, Maxime Lacroix, Lionel Arrive, Ziad Mallat, Soraya Taleb, Marc Diedisheim, Clément Cochain, Pierre-Emmanuel Rautou, Hafid Ait-Oufella

**Affiliations:** 1 Université Paris Cité, Inserm, PARCC, F-75015, Paris, France; 2 Service de médecine vasculaire, Hopital Européen G. Pompidou, Paris, France; 3 Service de gériatrie, Hopital Européen G. Pompidou, Paris, France; 4 Centre de recherche sur l’inflammation, Inserm, Paris, France; 5 Université de Bordeaux, UMR1034, Inserm, Biology of Cardiovascular Diseases, CHU de Bordeaux, Laboratoire d’Hématologie, Pessac, France; 6 Laboratoire d’Hématologie, Hôpital Saint-Antoine, AP-HP, Paris, France; 7 Institute of Experimental Biomedicine, University Hospital Wuerzburg, Würzburg, Germany; 8 Helmholtz Institute for RNA-based Infection Research (HIRI), Helmholtz-Center for Infection Research (HZI), Würzburg, Germany; 9 Service de radiologie, Hôpital Saint-Antoine, AP-HP, Paris, France; 10 GlandOmics, 41700 Cheverny, & Department of Diabetology, AP-HP, Hôpital Cochin, Paris, France; 11 AP-HP, Hôpital Beaujon, Service d’Hépatologie, DMU DIGEST, Centre de Référence des Maladies Vasculaires du Foie, FILFOIE, ERN RARE-LIVER, Clichy, France; 12 Medical Intensive Care Unit, Hôpital Saint-Antoine, AP-HP, Sorbonne Université, Paris, France

**Keywords:** Innate immune cells, Chronic inflammation, Cardiovascular biology

Correction to: *Nature Communications* 10.1038/s41467-022-34469-1, published online 03 November 2022

In this article the affiliation details for Giuseppe Rizzo were incorrectly given as Institute of Experimental Biomedicine, University Hospital Wuerzburg, Germany and Helmholtz Institute for RNA-based Infection Research (HIRI), Helmholtz-Center for Infection Research (HZI); Würzburg, Germany but should have been Institute of Experimental Biomedicine, University Hospital Wuerzburg, Würzburg, Germany.

Alma Zernecke was incorrectly given as Helmholtz Institute for RNA-based Infection Research (HIRI), Helmholtz-Center for Infection Research (HZI); Würzburg, Germany but should have been Institute of Experimental Biomedicine, University Hospital Wuerzburg, Würzburg, Germany.

Clément Cochain was incorrectly given as Institute of Experimental Biomedicine, University Hospital Wuerzburg, Germany and Helmholtz Institute for RNA-based Infection Research (HIRI), Helmholtz-Center for Infection Research (HZI); Würzburg, Germany but should have been Institute of Experimental Biomedicine, University Hospital Wuerzburg, Würzburg, Germany.

The original article has been corrected.

