# Supplementary material for: JAK2V617F mutation drives vascular resident macrophages toward a pathogenic phenotype and promotes dissecting aortic aneurysm
Source: Nat Commun. 2022 Nov 3;13:6592. doi: 10.1038/s41467-022-34469-1 (PMC9633755; doi:10.1038/s41467-022-34469-1)
Supplement: Supplementary file 1 — Supplementary Information [file 41467_2022_34469_MOESM1_ESM.pdf]

Parameters	Control patients N=157	JAK2V617F+ patients N=157	P value
**Age, years (mean +/- sd)**	64 +/-14	65 +/- 14	0.84
**Male (n, %)**	96 (61%)	96 (61%)	1
**Female (n, %)**	61 (39%)	61 (39%)	
**Hypertension (n, %)**	80 (51%)	68 (43%)	0.65
**Smoker (n, %)**	36 (23%)	58 (37%)	0.007
**Diabetes (n, %)**	17 (11%)	28 (18%)	0.08
**Diagnosis**			
**-Polycythemia vera**		43 (28%)	
**-Essential thrombocytemia**		53 (34%)	
**-Primary myelofibrosis**		19 (12%)	
**-Mixed myeloproliferative dis.**		22 (14%)	
**-Others**		20 (12%)	
**Treatment received**			
**-Hydroxyurea**		92 (59%)	
**-Ruxolotinib**		27 (17%)	

Supplementary Table 1. Characteristics of included patients. Comparisons between groups have been done using student t test or Chi-squared test.

Supplementary figure 1

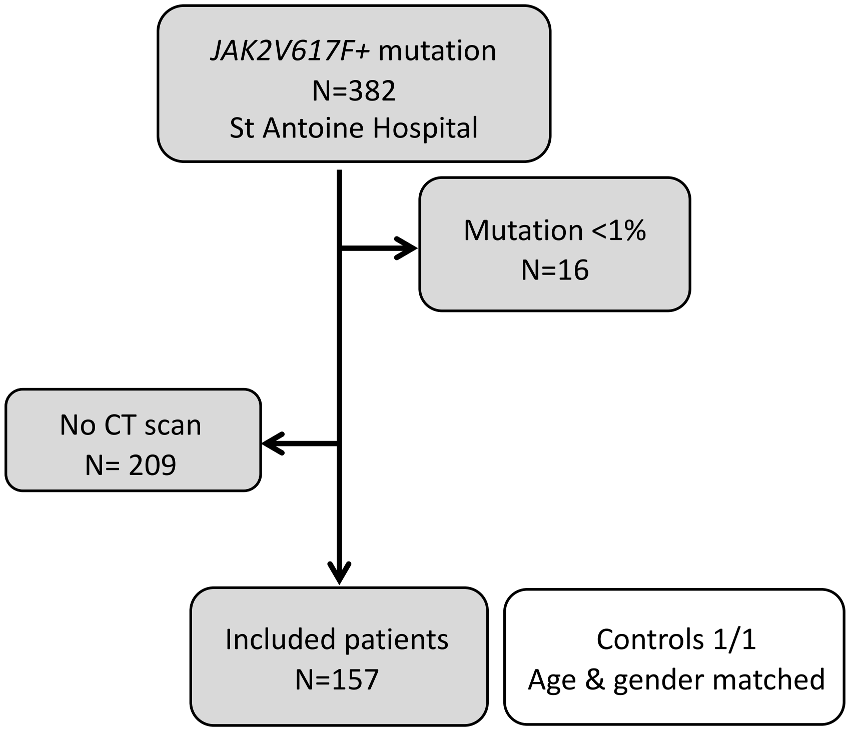

Supplementary figure 1. Flow chart of included patients.

Supplementary figure 2

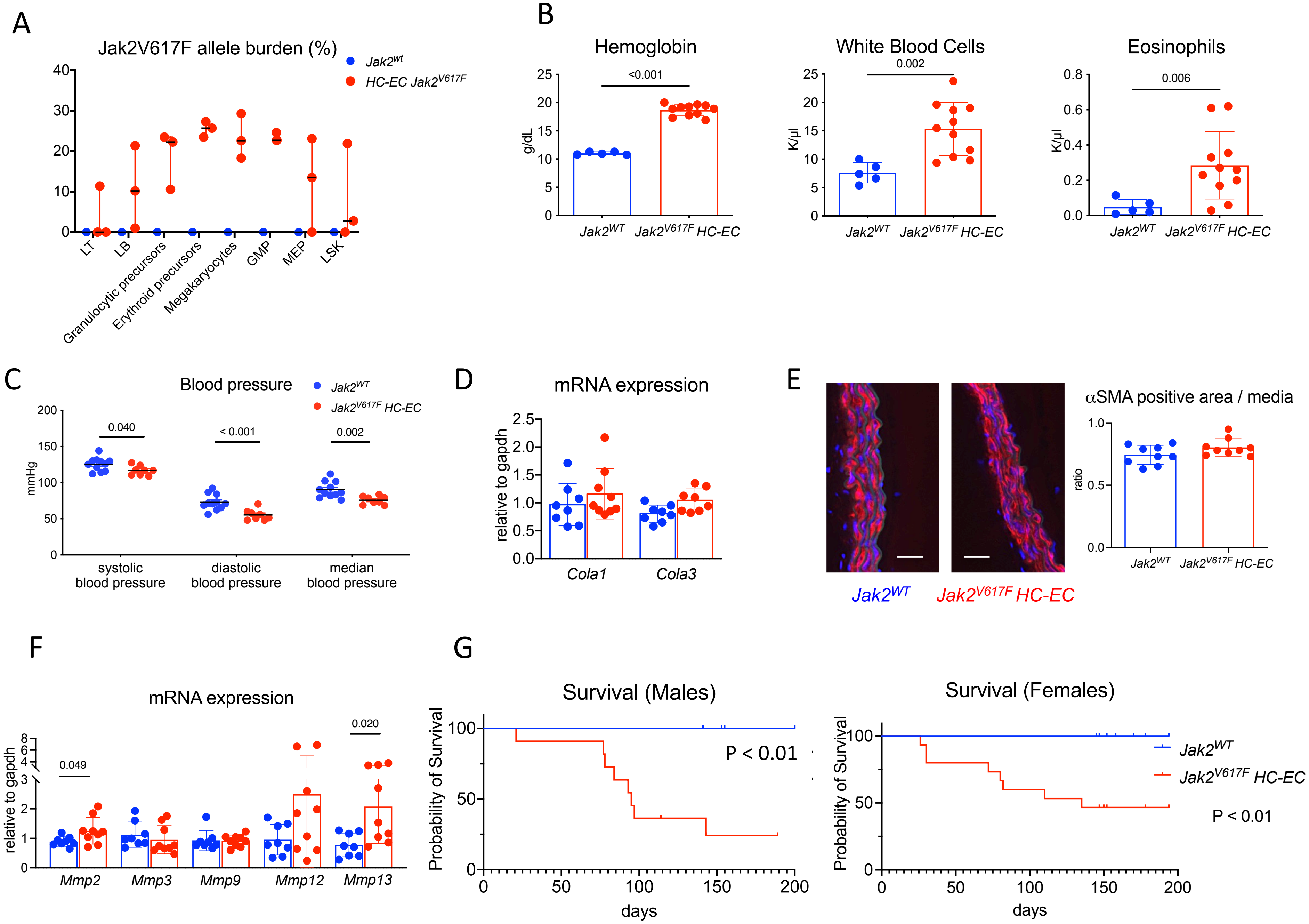

Supplementary figure 2: **A**, characterization of Jak2V617F mutation in stem cells and progenitors in the bone marrow of 7-week-old control *Jak2^{WT} and Jak2^{V617F} HC-EC* mice (N=3/group). **B**, Hemoglobin level, white blood cell and eosinophil cell count of 7-week-old control *Jak2^{WT}(N=5) and Jak2^{V617F} HC-EC* mice (N=11). **C**, Blood pressure measured on 7-week old *Jak2^{WT} (N=11) and Jak2^{V617F} HC-EC* mice (N=8). **D**, Cola1 and Cola3 mRNA expression in the aorta. **E**, representative photomicrographs and quantification of alpha-SMA content (immunostaining) in the aortic wall in 7-week-old animals (N=9/group), scale bar 50 µm. **F**, quantification of metalloprotease trascripts i, the aorta. **G**, Survival curves in male (N=6 *Jak2^{WT}* and N=11 *Jak2^{V617F}HC-EC*) and female (N=10 *Jak2^{WT}* and N=15 *Jak2^{V617F}HC-EC*) mice. *, P<0.05; **, P<0.01; ***<0.001. Source data are provided as a Source Data file.

Supplementary figure 3

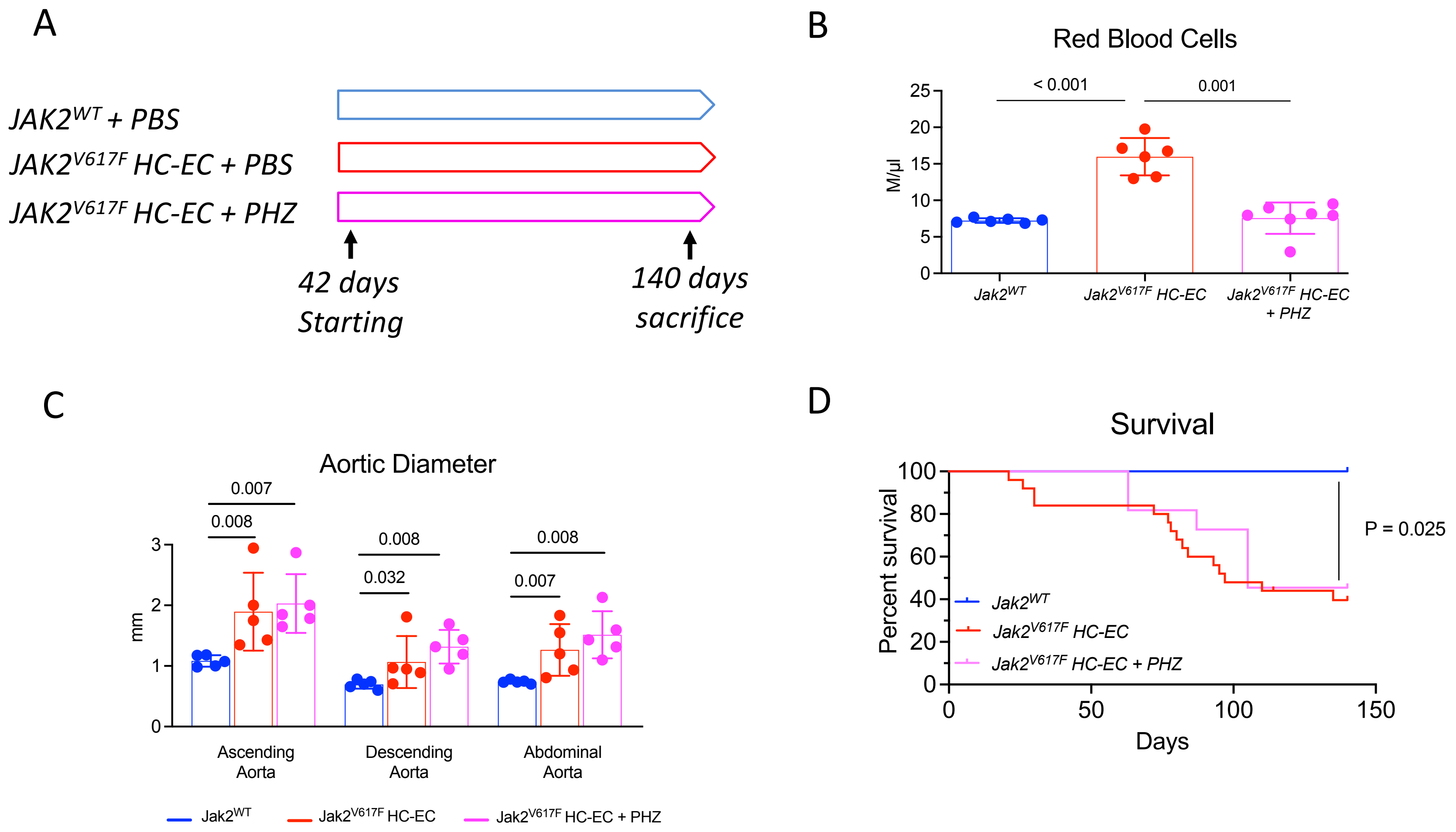

Supplementary figure 3: **Polyglobulia correction did not reduce aortopathy severity in JAK2 mutant mice. A**, experimental protocol, mice were treated either by PBS or by phenylhydrazine (PHZ) (25 mg/kg body weight every 3 days) (*Jak2^WT^* N=6, *Jak2^V617F^ HC-EC* + PBS and *Jak2^V617F^ HC-EC* + PHZ N=9). **B**, Red blood count after 2 months of treatment (*Jak2^WT^* N=6, *Jak2^V617F^ HC-EC* + PBS and *Jak2^V617F^ HC-EC* + PHZ N=7). **C**, quantitative analysis of the mean aortic diameter in surviving mice (N=5/group) in the thoracic (ascending and descending) and the abdominal aorta. **D**, Survival curves in *Jak2^WT^* (N=8) and *Jak2^V617F^HC-EC* (N=25) and *Jak2^V617F^HC-EC + PHZ* (N=11) *, P<0.05, **, P<0.01. Source data are provided as a Source Data file.

Supplementary figure 4

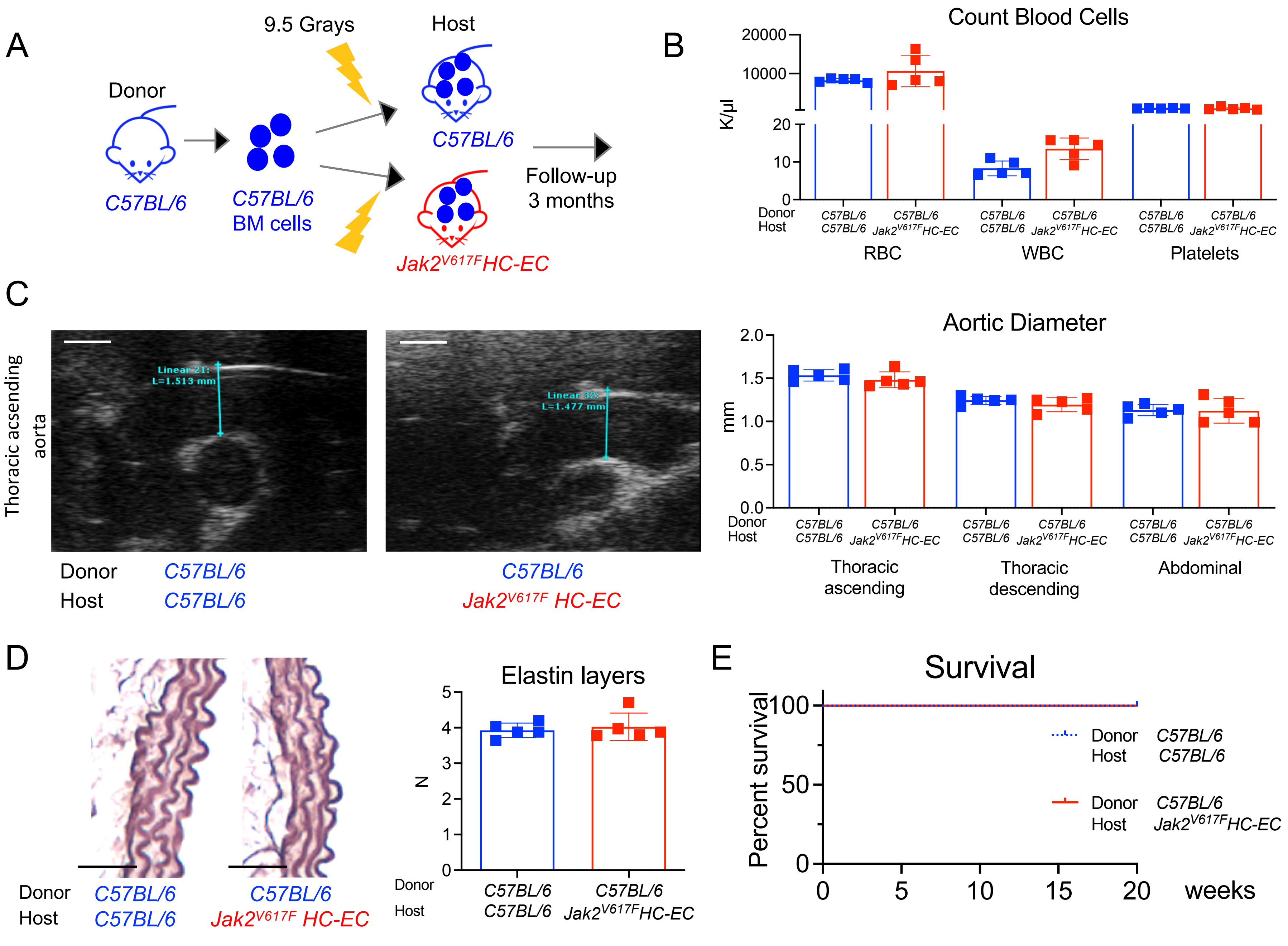

Supplementary figure 4: **JAK2V167F mutation in vascular cells did not promote dissecting aortopathy**

A, experimental protocol of WT bone marrow cell transplantation in lethally irradiated control *Jak2*^{WT} (N=5) and *Jak2*^{V617F} *HC-EC* mice (N=5). **B**, Red blood cell (RBC), white blood cell (WBC) and platelet count of 20-week-old chimeric mice (N=5/group). **C**, representative ultrasound photomicrographs and quantitative analysis of the mean aortic diameter in chimeric mice (N=5/group) in the thoracic (ascending and descending) and the abdominal aorta; scale bar 1mm. **D**, quantification of the number of elastin layers in the aortic wall by orcein staining (N=5/group), scale bar 50 µm. **E**, Survival curve (N=5/group). BM, bone marrow. Source data are provided as a Source Data file.

Supplementary figure 5

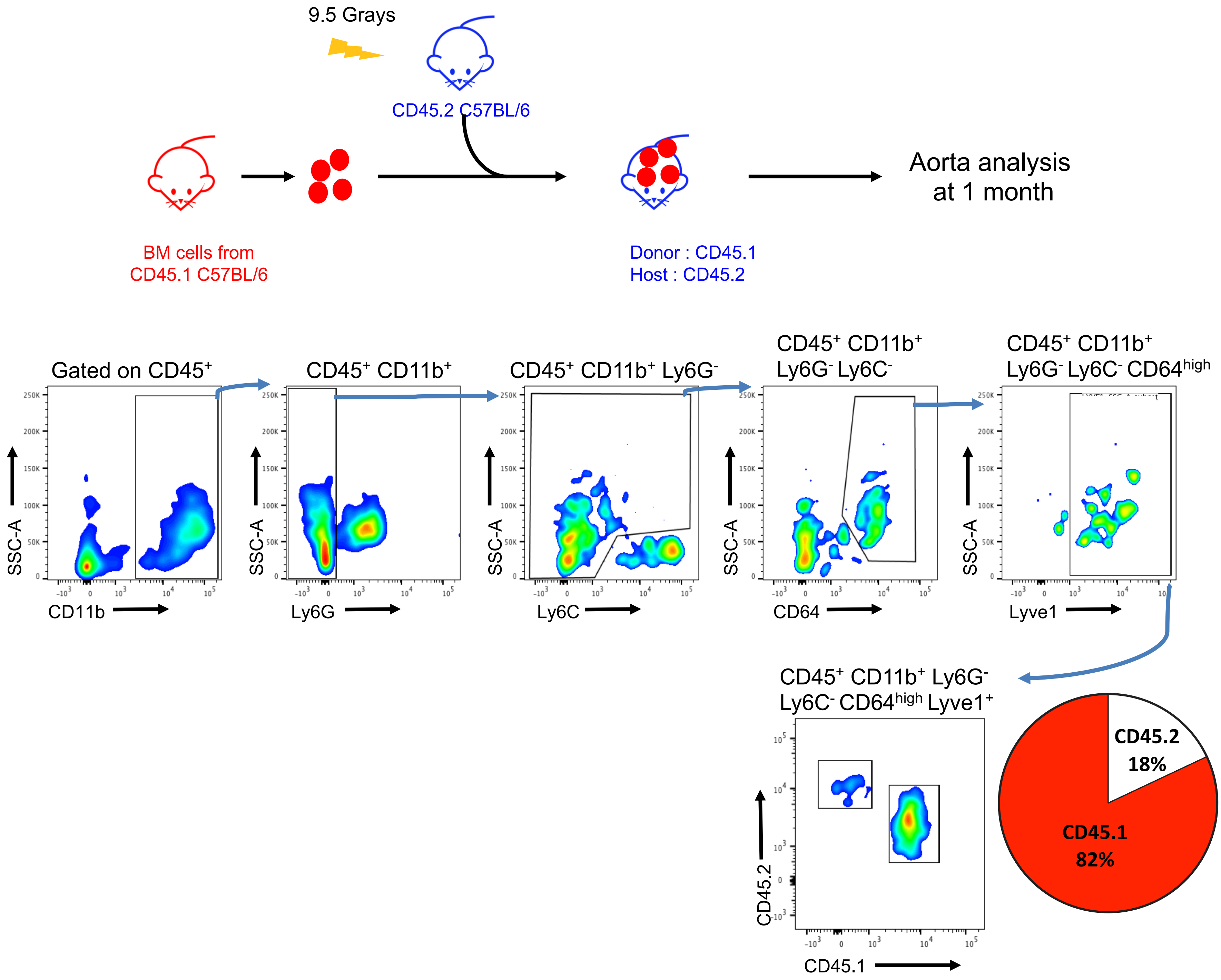

Supplementary figure 5. After lethal irradiation and BM transplantation most of vascular tissue resident macrophages derived from BM cells. C57BL/6 male CD45.2 mice (N=6) were irradiated (9.5 Gray) and retransplanted with CD45.1 BM cells. Four weeks after BMT, flow cytometry was performed on aortas. Neutrophils were defined as CD45+CD11b+Ly6G+ cells, monocytes as CD45+CD11b+Ly6G-Ly6C+ cells, macrophages as CD45+Ly6G-Ly6C-CD64+ cells and resident macrophages as CD45+Ly6G-Ly6C-CD64+Lyve1+ cells. BM, bone marrow.

Supplementary figure 6

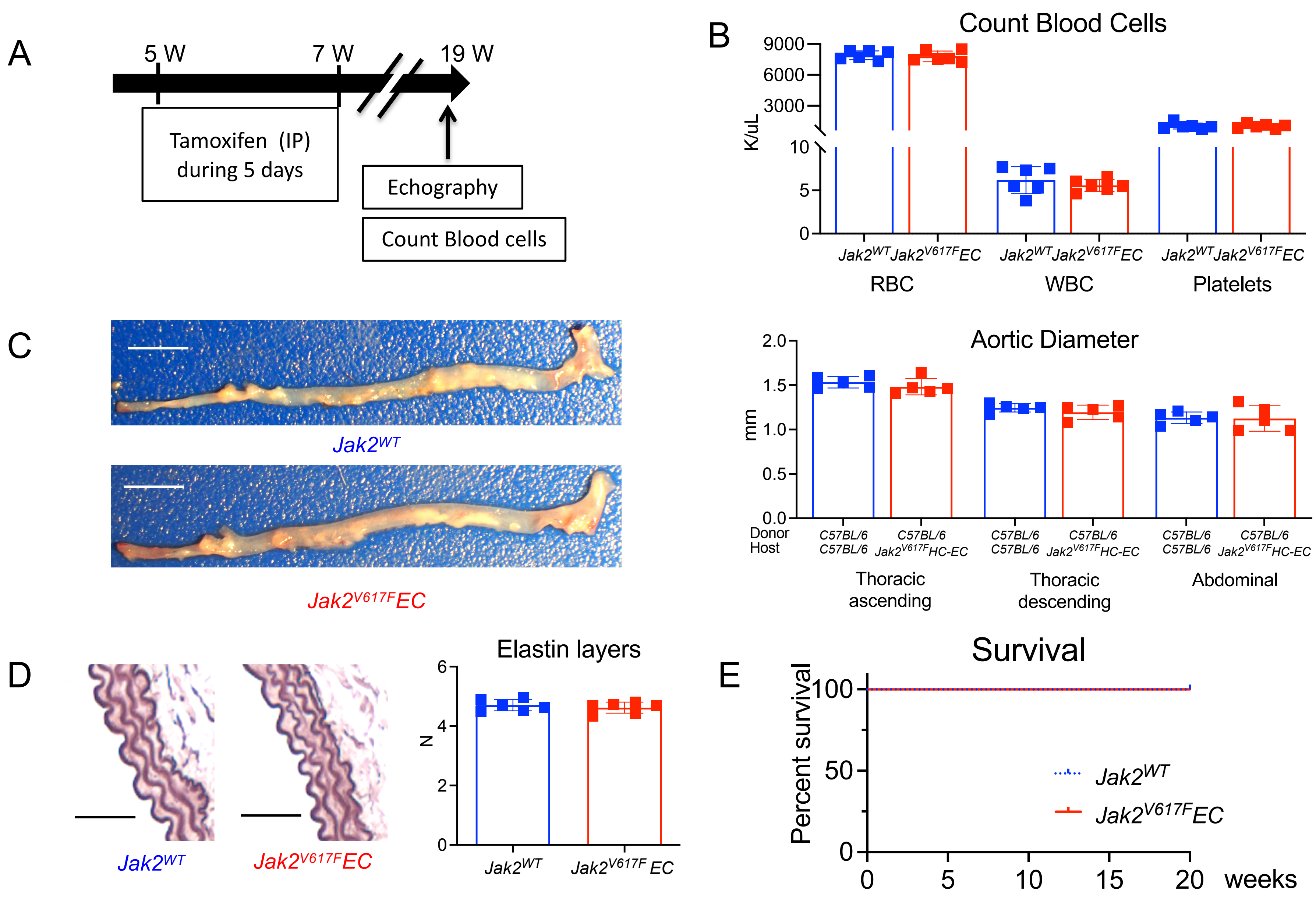

Supplementary figure 6: **A**, experimental protocol, tamoxifen induced selective expression of JAK2V617F mutation in endothelial cells in *Jak2*^{V617F} *EC* mice. **B**, Red blood cell, white blood cell and platelet count of 19-week-old *Jak2*^{WT} (N=6) and *Jak2*^{V617F} *EC* mice (N=6). **C**, representative photomicrographs and quantitative analysis of the mean aortic diameter in 19-week-old *Jak2*^{WT} and *Jak2*^{V617F} *EC* mice in the thoracic (ascending and descending) and the abdominal aorta, (N=6/group). scale bar 2 mm. **D**, quantification of the number of elastin layers in the aortic wall by Orcein staining (N=6/group), scale bar 50 µm. **E**, survival curve (N=24 control and N=13 *Jak2*^{V617F} *EC*). W, week. Source data are provided as a Source Data file.

Supplementary figure 7

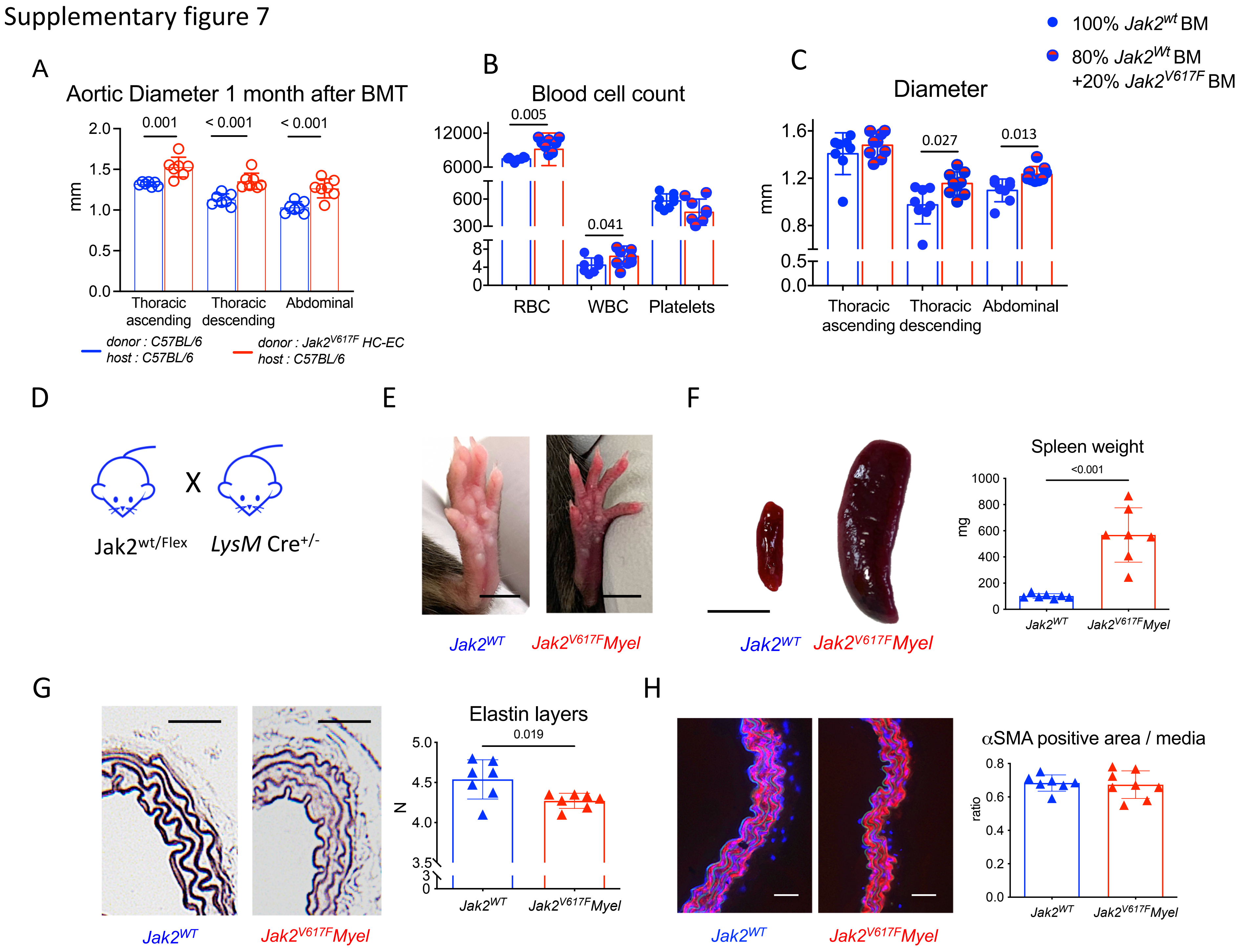

Supplementary figure 6: **A**, Quantitative analysis of the mean aortic diameter of chimeric mice one month after BMT. Irradiated C57BL/6 mice received either WT or *Jak2^{V617F} HC-EC* bone marrow cells (N=7/group). **B**, red blood cell, white blood cell and platelet count of chimeric C57BL/6 mice one month after irradiation and transfer of *Jak2^{WT}* BM cells or *80% Jak2^{WT}/20% Jak2^{V617F} HC-EC* BM cells (N=8/ group). **C**, ultrasonography quantification of aorta diameter one month after BMT (N=8/group). **D**, Generation of *Jak2^{V617F} Myel* strain. **E**, Paw pictures of 7-week-old Jak2^{WT} control or *Jak2^{V617F} Myel* mice showing palmar erythema in mutant mice, scale bar 1 cm. **F**, quantitative analysis and representative photomicrographs of spleen size in 7-week-old control Jak2^{WT} and *Jak2^{V617F} Myel* mice (N=7/group) (scale bar 1cm). **G**, representative photomicrographs and quantification of the number of elastin layers in the aortic wall by orcein staining in 7-week old animals (N=7/group), scale bar 50 µm. **H**, representative photomicrographs and quantification of alpha-SMA content (immunostaining) in the aortic wall in 7-week old animals (N=7 in control group and N=8 in *Jak2^{V617F} Myel* group), scale bar 50 µm. *, P<0.05, **, P<0.01, ***, P<0.001. BMT, Bone marrow transplantation. Source data are provided as a Source Data file.

Supplementary figure 8

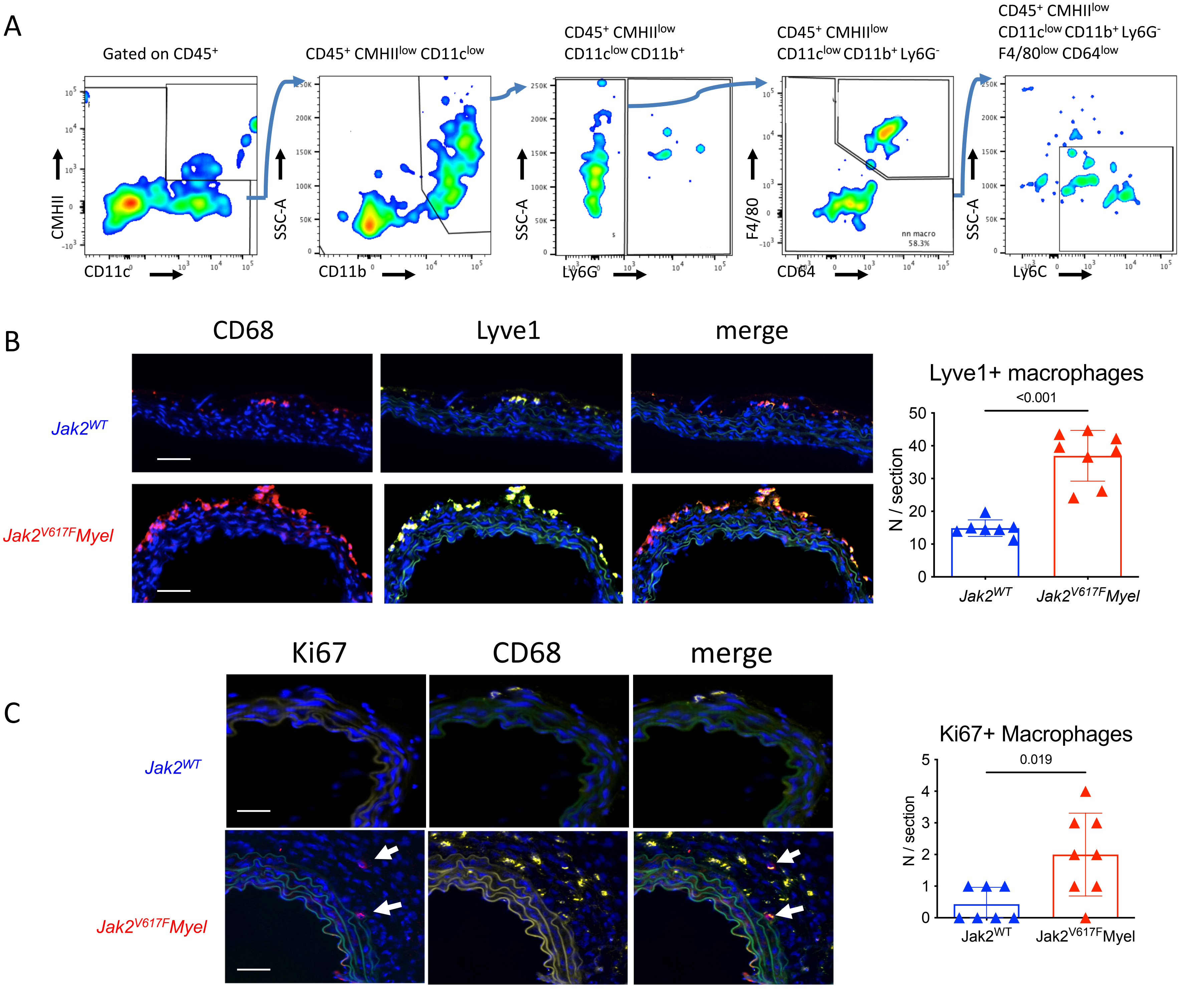

Supplementary figure 8. **A**, Gating strategy to identify CD45^{+}CD11c^{low}MHCII^{Low}CD11b^{+}Ly6G^{+} neutrophils, and CD45^{+}CD11c^{low}MHCII^{Low}CD11b^{+}Ly6G^{-}CD64^{+}F4/80^{+} macrophages and CD45^{+}CD11c^{low}MHCII^{low}CD11b^{+}Ly6G^{-}CD64^{low}F4/80^{low}Ly6C^{+} monocytes in the aortic wall by flow cytometry. **B**, representative photomicrographs and quantification of the number of CD68+ macrophages colocalizing with Lyve1 staining in the aortic wall (immunostaining) in 7-week-old animals (N=7 in control group and N=8 in *Jak2^{V617F} Myel* group), scale bar 50 µm. **C**, representative photomicrographs and quantification of CD68+ macrophages colocalizing with Ki67 staining (immunostaining) in the aortic wall in 7-week-old animals (N=7 in control group and N=8 in *Jak2^{V617F} Myel* group), scale bar 50 µm. *, P<0.05, **, ***, P<0.001. Source data are provided as a Source Data file.

Supplementary figure 9

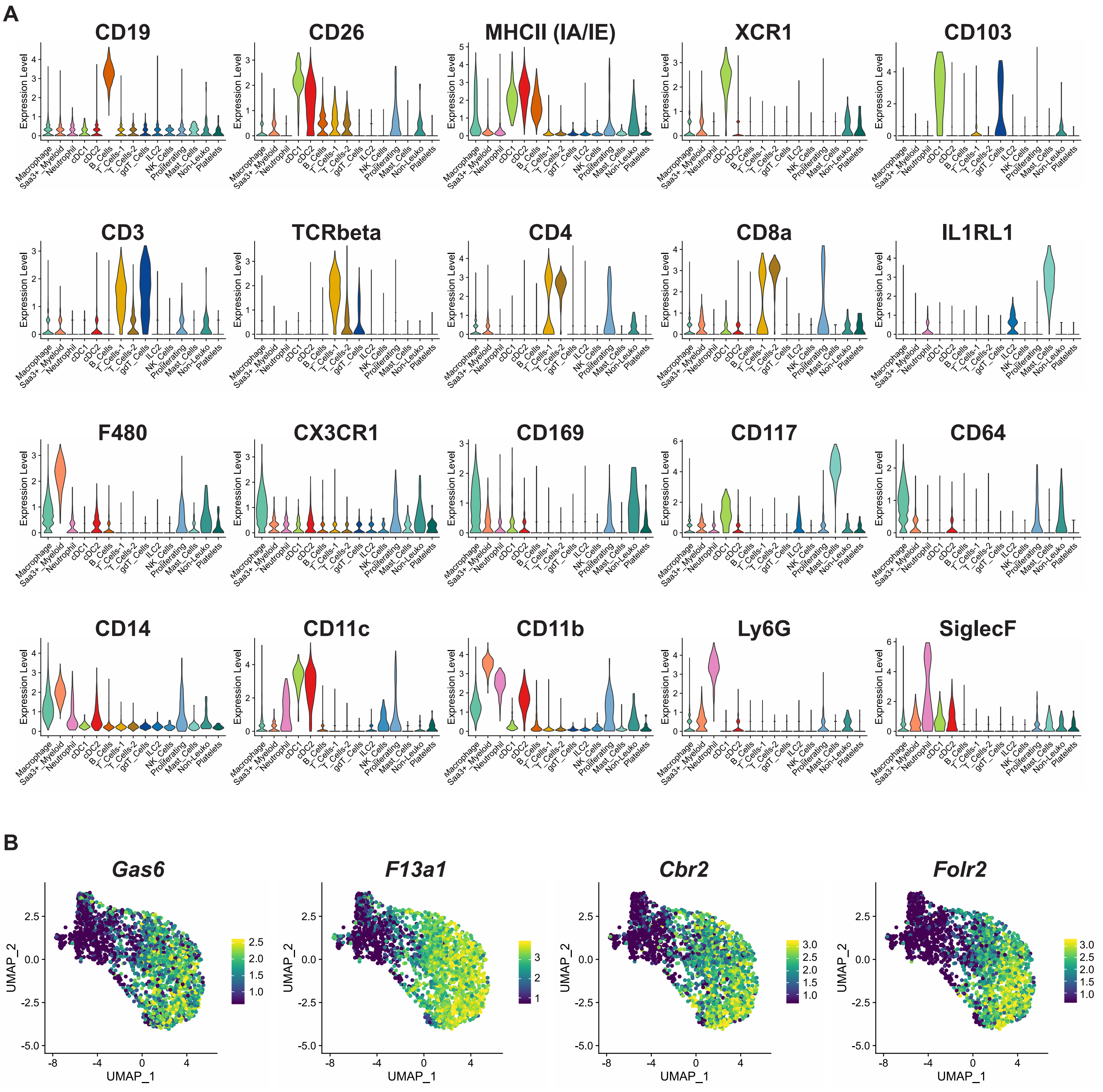

Supplementary figure 9. Additional single-cell RNA-seq/CITE-seq analysis. A, expression of the indicated cell surface markers measured by CITE-seq in total CD45+ cells allowed identification of major immune lineages in aortas; **B,** expression of the indicated tissue resident macrophage marker transcripts projected on the UMAP plot of aortic monocyte/macrophages. UMAP, Uniform Manifold Approximation and Projection for Dimension Reduction; CITE-seq, Cellular Indexing of Transcriptomes and Epitopes by Sequencing

Supplementary figure 10

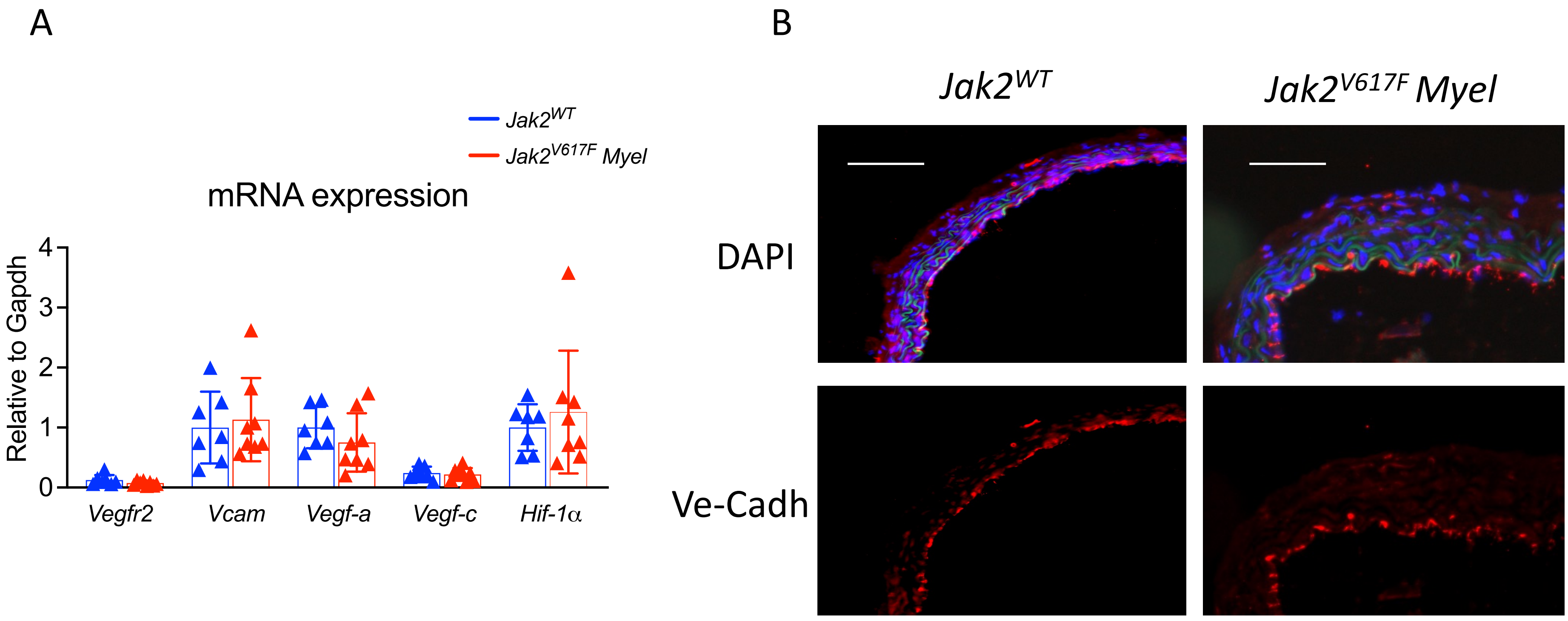

Supplementary figure 10. Jak2V617F mutation did not impair angiogenic pathways in the aortic wall. A, quantification of *Vegfr2, Vegfa, Vegfc* and *Hifa* transcripts by qPCR in the aorta of 8-week-old *Jak2^{WT}* control and *Jak2^{V617F} Myel* mice. **B**, Immunofluorescent staining of Ve-Cadherin in the aortic wall of of 8-week-old *Jak2^{WT}* control and *Jak2^{V617F} Myel* mice. scale bar 50 µm. Source data are provided as a Source Data file.

Supplementary figure 11

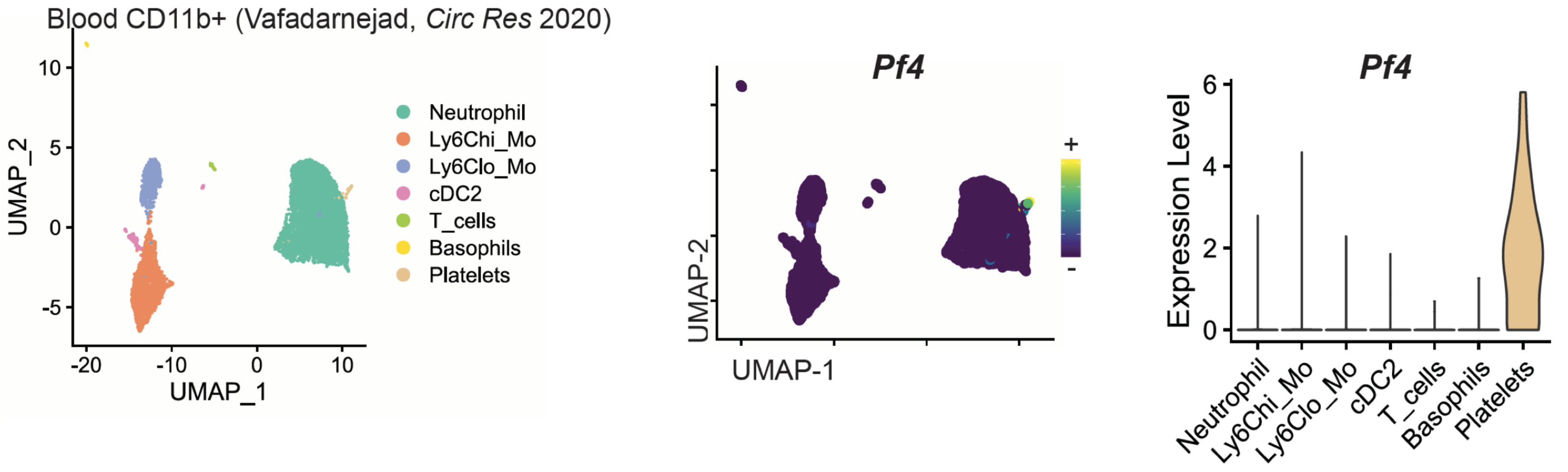

Supplementary figure 11. UMAP plot of mouse blood CD11b+CD19-NK1.1-Ter119- cells with identification of cell types (left) and expression of Pf4 projected on the UMAP plot (right). UMAP, Uniform Manifold Approximation and Projection for Dimension Reduction; Pf, platelet factor.

Supplementary figure 12

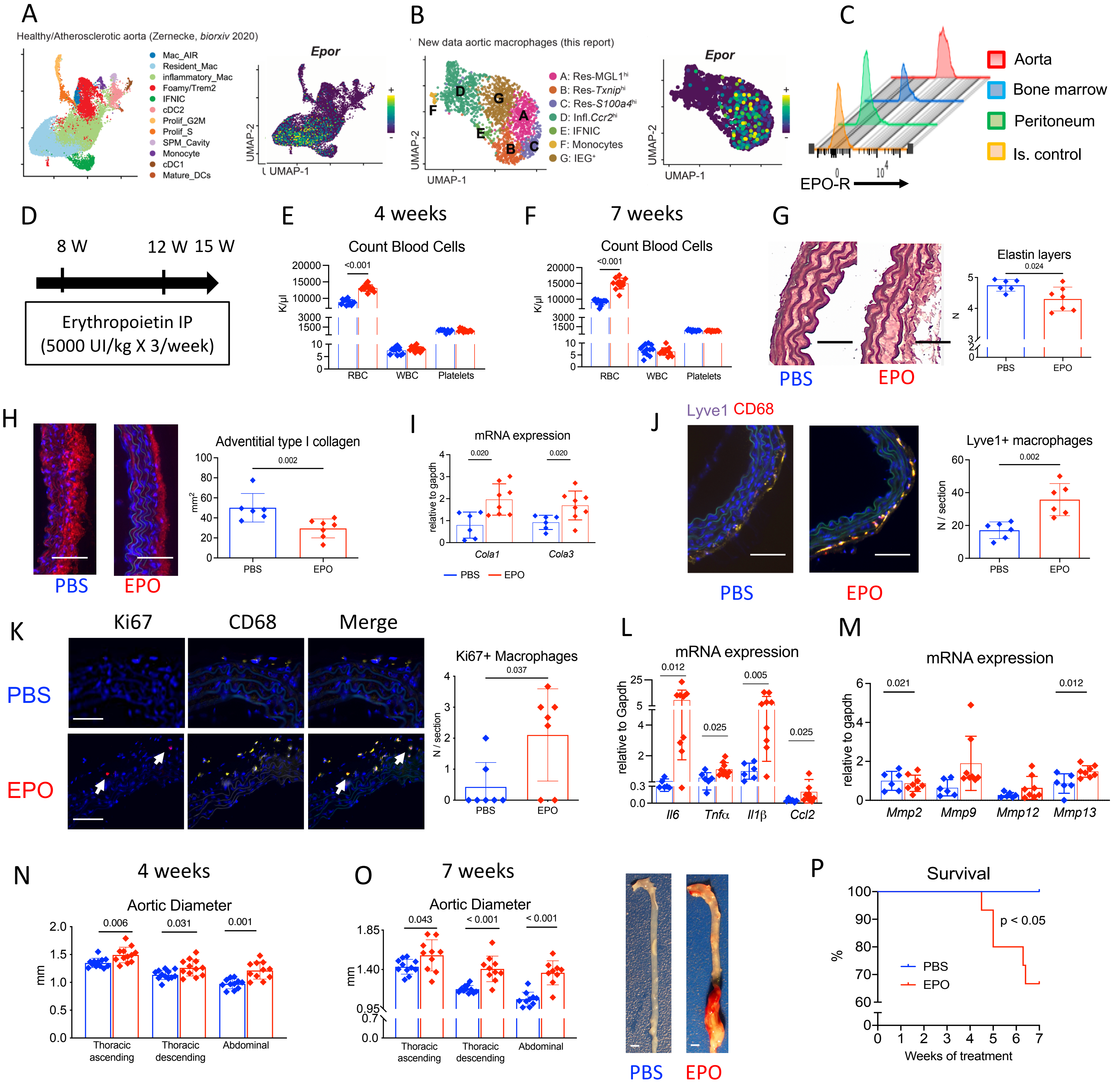

Supplementary figure 12. A, left cells corresponding to monocytes/macrophages/dendritic cells were extracted from murine healthy and atherosclerotic plaques and separately reanalyzed with dimensional reduction (UMAP, [23]). Right, expression of EpoR transcript projected on the vascular monocytes/macrophages/dendritic cells UMAP plot. **B**, left, cells corresponding to macrophages were extracted from murine aorta and separately reanalyzed with dimensional reduction (UMAP). Right, expression of EpoR transcript projected on the vascular tissue resident macrophage subsets UMAP plot. **C**, Analysis by flow cytometry of the EPO-R expression by macrophages isolated from the aorta (Red), the bone marrow (Blue), the peritoneum (Green). **D**, experimental protocol of recombinant Erythropoietin (EPO) supplementation in male C57BL/6 mice. **E**, **F**, red blood cell, white blood cell and platelet count of C57BL/6 mice treated by PBS or EPO during 4 and 7 weeks (N=8/Group/time). Histological analysis was done after 4 weeks of treatment. **G**, quantification of the number of elastin layers in the aortic wall by Orcein staining (N=7/group), scale bar 50 μm. **H**, Quantification of the collagen content (Cola1 immunostaining) in the aortic wall at sacrifice (N=7/group), scale bar 50 μm. **I**, quantification of Cola1 and Cola3 mRNA expression by RT-qPCR in the aorta of PBS or EPO-treated mice (N=6 in PBS group and N=8 in EPO group). **J**, quantification of Lyve-1+ CD68+ tissue resident macrophages in the abdominal aorta at sacrifice (Immunostaining, N=7/group), scale bar 50 µm. **K**, representative photomicrographs and quantification of CD68+ macrophages colocalizing with Ki67 staining (immunostaining) in the aortic wall of PBS or EPO-treated C57BL/6 animals (N=7/group), scale bar = 50 μm. **L**, Quantification of *Il6, Tnf*α*, Il1*β *and Ccl2* mRNA expression by RT-qPCR in the aorta of PBS (N=6) or EPO-treated mice (N=9). **M**, quantification of Mmps mRNA expression by RT-qPCR in the aorta of PBS (N=6) or EPO-treated mice (N=8). **N**, **O**, representative photomicrographs and quantitative analysis of the mean aortic diameter in PBS or EPO-treated mice (N=11/ group at 4 weeks of treatment and N=10/group at 7 weeks of treatment) in the thoracic (ascending and descending) and the abdominal aorta, scale bar 1mm. **P**, Survival curve after 4 and 7 weeks of treatment. [Mann-Whitney test, *P<0.05, ** P<0.01]. UMAP, Uniform Manifold Approximation and Projection for Dimension Reduction; EPO, erythropoietin; *Il*, interleukin; *Tnf*, tumor necrosis factor. Source data are provided as a Source Data file.

Supplementary figure 13

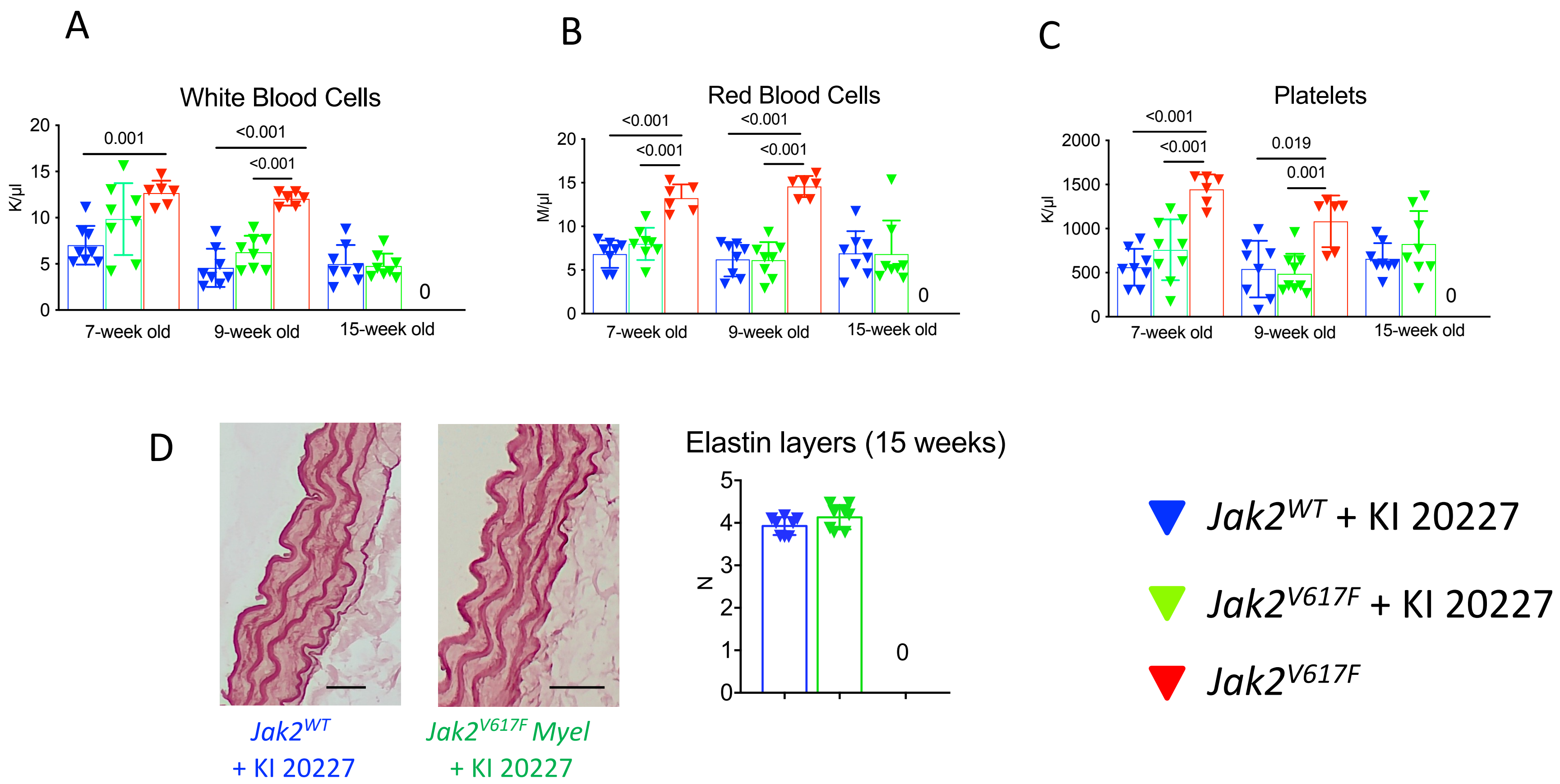

Supplementary figure 13. white blood cell (**A**), red blood cell (**B**) and platelet (**C**) count of male mice treated by PBS or KI20227. Protocol was started at 5 weeks of age until 15 weeks. Blood cells analysis was done at 7 weeks, 9 weeks in all animals and finally at 15 weeks in survivors. **D**, representative pictures and quantification of the number of elastin layers in the aortic wall by Orcein staining in surviving Ki20227-treated *Jak2*^{WT} (blue) and *Jak2*^{V617F} *Myel* (green) mice (N=8/group), scale bar 50 µm *, P<0.05, **, P<0.01. Source data are provided as a Source Data file.
