# Supplementary material for: JAK2V617F mutation drives vascular resident macrophages toward a pathogenic phenotype and promotes dissecting aortic aneurysm
Source: Nat Commun. 2022 Nov 3;13:6592. doi: 10.1038/s41467-022-34469-1 (PMC9633755; doi:10.1038/s41467-022-34469-1)
Supplement: Supplementary file 3 — Reporting Summary [file 41467_2022_34469_MOESM3_ESM.pdf]

nature portfolio

Corresponding author(s): Ait-Oufella

Last updated by author(s): Jul 21, 2022

Reporting Summary

Nature Portfolio wishes to improve the reproducibility of the work that we publish. This form provides structure for consistency and transparency in reporting. For further information on Nature Portfolio policies, see our Editorial Policies and the Editorial Policy Checklist.

Statistics

For all statistical analyses, confirm that the following items are present in the figure legend, table legend, main text, or Methods section.

n/a	Confirmed	
☐	☒	The exact sample size (n) for each experimental group/condition, given as a discrete number and unit of measurement
☐	☒	A statement on whether measurements were taken from distinct samples or whether the same sample was measured repeatedly
☐	☒	The statistical test(s) used AND whether they are one- or two-sided<br>*Only common tests should be described solely by name; describe more complex techniques in the Methods section.*
☐	☒	A description of all covariates tested
☐	☒	A description of any assumptions or corrections, such as tests of normality and adjustment for multiple comparisons
☐	☒	A full description of the statistical parameters including central tendency (e.g. means) or other basic estimates (e.g. regression coefficient) AND variation (e.g. standard deviation) or associated estimates of uncertainty (e.g. confidence intervals)
☐	☒	For null hypothesis testing, the test statistic (e.g. F, t, r) with confidence intervals, effect sizes, degrees of freedom and P value noted<br>*Give P values as exact values whenever suitable.*
☒	☐	For Bayesian analysis, information on the choice of priors and Markov chain Monte Carlo settings
☒	☐	For hierarchical and complex designs, identification of the appropriate level for tests and full reporting of outcomes
☒	☐	Estimates of effect sizes (e.g. Cohen's d, Pearson's r), indicating how they were calculated

Our web collection on statistics for biologists contains articles on many of the points above.

Software and code

Policy information about availability of computer code

Data collection	Material and methods, paragraph "human samples analysis" (page 21); paragraph "data availability" (Page 35)
Data analysis	Material and methods, paragraph "Bulk RNA sequencing and analysis" (page 28); paragraph "Single-cell RNA-seq data analysis" (page 32); paragraph "statistical analysis" (Page 34)

For manuscripts utilizing custom algorithms or software that are central to the research but not yet described in published literature, software must be made available to editors and reviewers. We strongly encourage code deposition in a community repository (e.g. GitHub). See the Nature Portfolio guidelines for submitting code & software for further information.

Data

Policy information about availability of data

All manuscripts must include a data availability statement. This statement should provide the following information, where applicable:

- Accession codes, unique identifiers, or web links for publicly available datasets
- A description of any restrictions on data availability
- For clinical datasets or third party data, please ensure that the statement adheres to our policy

Material and methods, data availability (Page 35)

Human research participants

Policy information about studies involving human research participants and Sex and Gender in Research.

Reporting on sex and gender	material and methods, paragraph "human samples analysis" (page 21); Supplementary figure 1B
Population characteristics	material and methods, paragraph "human samples analysis" (page 21); Supplementary figure 1B
Recruitment	material and methods, paragraph "human samples analysis" (page 21);
Ethics oversight	material and methods, paragraph "human samples analysis" (page 21);

Note that full information on the approval of the study protocol must also be provided in the manuscript.

Field-specific reporting

Please select the one below that is the best fit for your research. If you are not sure, read the appropriate sections before making your selection.

[x] Life sciences [ ] Behavioural & social sciences [ ] Ecological, evolutionary & environmental sciences

For a reference copy of the document with all sections, see nature.com/documents/nr-reporting-summary-flat.pdf

Life sciences study design

All studies must disclose on these points even when the disclosure is negative.

Sample size	Material and methods and figures ' legends
Data exclusions	NA
Replication	material and methods and figures'legends
Randomization	NA
Blinding	All analyses have been done blindly

Reporting for specific materials, systems and methods

We require information from authors about some types of materials, experimental systems and methods used in many studies. Here, indicate whether each material, system or method listed is relevant to your study. If you are not sure if a list item applies to your research, read the appropriate section before selecting a response.

Materials & experimental systems

n/a	Involved in the study	
[ ]	[x]	Antibodies
[x]	[ ]	Eukaryotic cell lines
[x]	[ ]	Palaeontology and archaeology
[ ]	[x]	Animals and other organisms
[ ]	[x]	Clinical data
[x]	[ ]	Dual use research of concern

Methods

n/a	Involved in the study	
[x]	[ ]	ChIP-seq
[ ]	[x]	Flow cytometry
[x]	[ ]	MRI-based neuroimaging

Antibodies

Antibodies used	Material and methods; paragraph "Flow cytometry" (page 25); paragraph "Determination of JAK2V617F burden" (page 26); paragraph "CITE-Seq" (Page 29-30)
Validation	Material and methods

Animals and other research organisms

Policy information about studies involving animals; ARRIVE guidelines recommended for reporting animal research, and Sex and Gender in Research

Laboratory animals	Material and methods; paragraph "murine models" (page 21)
Wild animals	NA
Reporting on sex	Material and methods; paragraph "murine models" (page 21)
Field-collected samples	NA
Ethics oversight	Material and methods; paragraph "murine models" (page 21), ethical committee approval (MESR26963)

Note that full information on the approval of the study protocol must also be provided in the manuscript.

Clinical data

Policy information about clinical studies

All manuscripts should comply with the ICMJEguidelines for publication of clinical research and a completedCONSORT checklist must be included with all submissions.

Clinical trial registration	Material and methods; paragraph "human samples analysis" (page 21);
Study protocol	Material and methods; paragraph "human samples analysis" (page 21);
Data collection	Material and methods; paragraph "human samples analysis" (page 21);
Outcomes	NA

Flow Cytometry

Plots

Confirm that:

- [x] The axis labels state the marker and fluorochrome used (e.g. CD4-FITC).
- [x] The axis scales are clearly visible. Include numbers along axes only for bottom left plot of group (a 'group' is an analysis of identical markers).
- [x] All plots are contour plots with outliers or pseudocolor plots.
- [x] A numerical value for number of cells or percentage (with statistics) is provided.

Methodology

Sample preparation	Material and methods, "Flow cytometry" (page 25)
Instrument	Material and methods, "Flow cytometry" (page 25);
Software	Material and methods, "Flow cytometry" (page 25);
Cell population abundance	Material and methods, "Flow cytometry" (page 25);
Gating strategy	Material and methods, "Flow cytometry" (page 25);

- [x] Tick this box to confirm that a figure exemplifying the gating strategy is provided in the Supplementary Information.
